# E-Health-based, trans-sectoral, geriatric health service – Geriatric Network (GerNe)

**DOI:** 10.1038/s41598-024-67624-3

**Published:** 2024-07-27

**Authors:** Michael Mohr, Matthias Büttner, Oliver Deuster, Jochen Heckmann, Frank Huwer, Irene Krämer, Cornelia Lippold, Bettina Siegrist, Susanne Singer, Marina Veith, Ariane Zinke, Roland Hardt

**Affiliations:** 1 Geriatric Department, University Medical Center of the Johannes Gutenberg University Mainz, Mainz, Germany; 2 Institute of Medical Biostatistics, Epidemiology and Informatics (IMBEI), University Medical Center of the Johannes Gutenberg University Mainz, Mainz, Germany; 3 Interdisciplinary Center for Clinical Trials (IZKS), University Medical Center of the Johannes Gutenberg University Mainz, Mainz, Germany; 4 Geriatric Clinic Rheinhessen-Nahe, Bad Kreuznach, Germany; 5 Pharmacy, University Medical Center of the Johannes Gutenberg University Mainz, Mainz, Germany; 6 St. Vincenz Hospital Diez, Diez, Germany; 7 St. Marien- und St. Annastifts Hospital Ludwigshafen, Ludwigshafen, Germany; 8 BARMER Rheinland-Pfalz/Saarland, Mainz, Germany

**Keywords:** Geriatric care, Case file, Elderly, Rehospitalization, Consil, Health care, Medical research

## Abstract

Currently, exchange of information between the geriatric clinic and the attending general practitioner (GP) occurs primarily through the doctor's letter after discharging from the clinic. The aim of our study was to reduce readmissions of multimorbid, geriatric patients to the clinic by establishing a new form of care via an electronic case file (ECF) and a consultation service (CS). The discharging geriatric clinic filled out an online ECF. The patient's GP should document quarterly follow-ups in the ECF. The case file was monitored by the discharging clinic due to a consultation service. The primary efficacy endpoint was the rehospitalization rate within one year. The hospitalization rate for patients managed in the project was 83.1/100 person years (PY), while the control group from insurance data had a rate of 69.0/100 PY. The primary endpoint did not show a statistically significant difference (p = 0.15). A total of 195 contacts were documented via CS for 171 participants, mostly initiated by the clinics. The clinical queries primarily concerned drug therapy. The Covid pandemic had an overall impact on hospitalizations. There are many approaches to reducing hospital readmissions after discharge of older patients. Supporting the transition from inpatient to outpatient care by different professional groups or care systems has been shown to have a positive effect. Furthermore, the utilisation of an ECF can also be beneficial in this regard.

## Introduction

Due to demographic developments and increasing life expectancy in Germany, the proportion of older patients, particularly those aged 80 years and above, is expected to rise in the coming years. Currently approximately 7% (6.2 million people) of the population in Germany is 80 years and older. In 2040 this proportion is expected to be approximately 9% (7.4 million people). Subsequently the healthcare system has to stay abreast of these changes with a growing number of older and multimorbid patients (presence of several chronic diseases)^1^.

The milestone study by Rubenstein et. al. already showed a superiority of the geriatric therapy approach over standard care^2,3^. The geriatric therapy approach aims to maintain or improve the functional abilities of older adults through a multidisciplinary team of healthcare professionals (including doctors, physiotherapists, occupational therapists, specially trained nursing staff, social workers). It focuses on function-oriented goals, prevention of complications, personalized treatment plans, and long-term support to enhance independence and quality of life. This approach aims to meet the specific requirements and difficulties faced by older individuals, enhancing their general welfare and decreasing the likelihood of hospitalisation and dependence on long-term care^4^. An important element in the management of geriatric patients is the performance of a Comprehensive Geriatric Assessment (CGA). This is a multidimensional, interdisciplinary diagnostic procedure used for the comprehensive assessment of older people. It aims to assess the health status, functional capacity, psychosocial situation and quality of life of older people. Various domains such as medical, functional, cognitive, psychological and social aspects are considered^4–6^. Rubenstein's 1984 study found that patients treated in a specialized geriatric unit had a lower mortality rate and were significantly less likely to be admitted to nursing homes than the comparison group of patients receiving standard care. For this reason, the treatment of geriatric patients should be directed towards a specialized department^2^.

In Germany, the number of geriatric cases is expected to increase by 33% in the coming years. While the inpatient sector is increasingly specializing in the treatment of geriatric and multimorbid patients and focusing on geriatric care, the outpatient care sector for geriatric patients continues to face a major challenge. In addition to the complexity and time-consuming nature of geriatric care, there are other factors that can occasionally hinder optimal care of elderly patients in outpatient settings. One major issue is the transition from inpatient to outpatient care. Information loss often occurs when geriatric patients are discharged from the clinic to primary care. The exchange of information between the discharging clinic and the primary care physician, who continues the treatment in the patient's home environment, only takes place unidirectionally via the doctor's letter. The German Geriatric Society conducted a survey that identified 31 projects examining the transition from inpatient geriatric treatment to an outpatient setting. Depending on the project, the transition was accompanied by various professional groups, including medical services, nursing, therapeutic teams, nutritionists, pharmacy and social services^7^. Transitional care models (TCM) have the potential to prevent readmissions of older people^8,9^. Positive effects have already been observed for geriatric co-management when accompanied by physicians and/or specialized nursing staff as part of the discharge process for older adults^10,11^. Additionally, it is important to acknowledge the beneficial impact of family involvement on the discharge process^12^.

The GerNe project aims to enhance geriatric patient care by implementing a cross-sectoral care approach. This approach involves the establishment of an electronic case file (ECF) to reduce the rate of hospital readmissions following an inpatient stay in a geriatric clinic. The results of the GerNe project can make a contribution to the digitalization of the German healthcare system and provide potential uses in the context of the introduction of an electronic patient file (ePA)^13^.

## Methods

### Study design and participants

A prospective, multicentre, epidemiological study was performed in a mixed-method approach with the use of secondary data. The study was conducted at four geriatric specialty hospitals in Rhineland-Palatinate, Germany. Inclusion criteria were completion of an acute geriatric complex treatment in one of the participating clinics, age > 70 years, geriatric multimorbidity and life expectancy > 12 months (Fig. 2). Patients in the intervention group were recruited at one of the participating hospitals and received the new form of care. The control group was provided from the data of the BARMER health insurance, a statutory health insurance provider in Germany (Scientific Data Warehouse (W-DWH)). Written informed consent was obtained from all patients to participate in the study. In the case of patients unable to consent, informed consent was obtained from a legal guardian.

### New form of care

**Figure 1:**
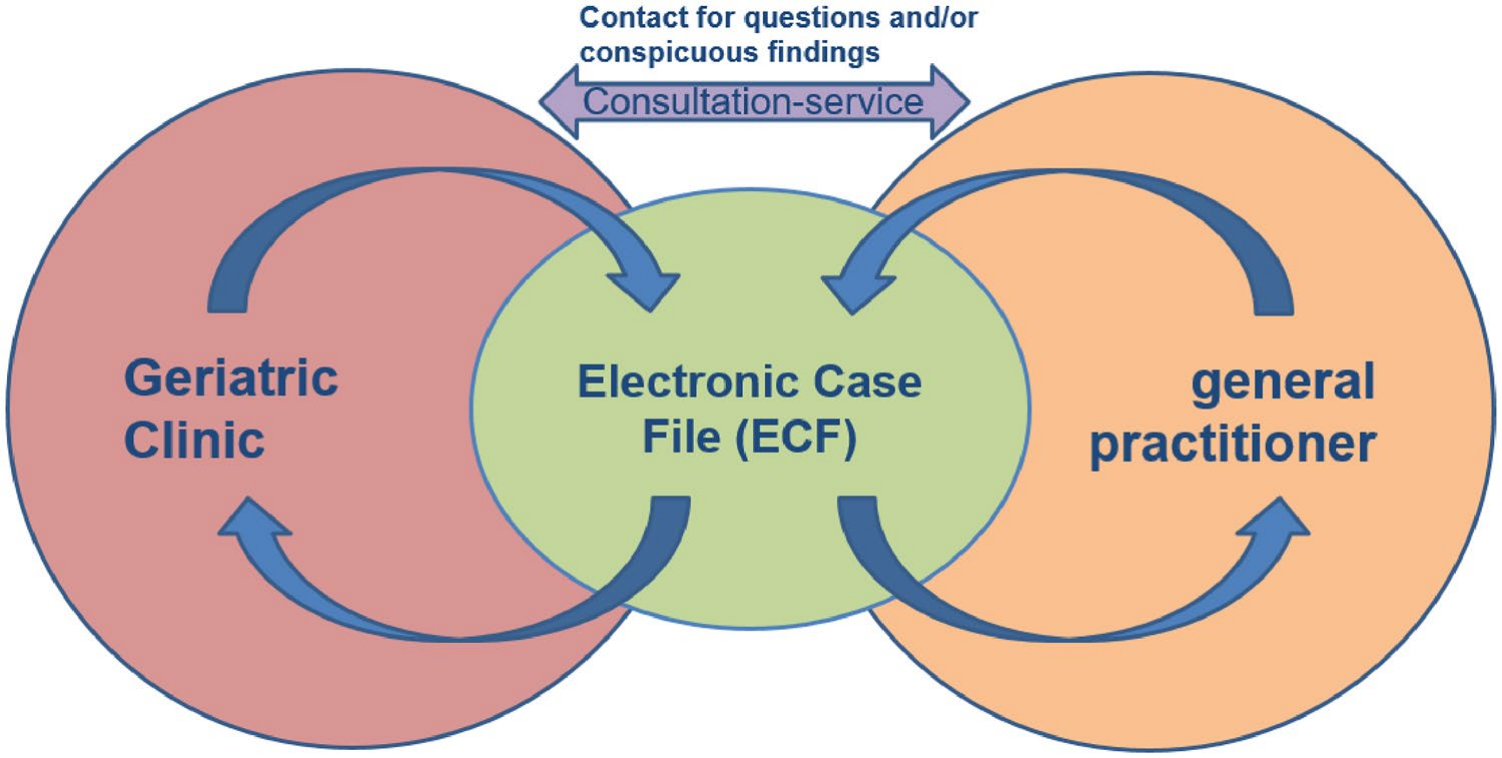
Bidirectional exchange within the framework of the new form of care.

The new form of care centers around a web-based electronic case file (ECF), which acts as a communication platform between the geriatric clinic and participating general practitioners (GPs). Additionally, a consultation service has been implemented to address any questions from the treating GPs and the clinic, as well as to monitor the case file for medical plausibility (see Fig. 1).

**Figure 2:**
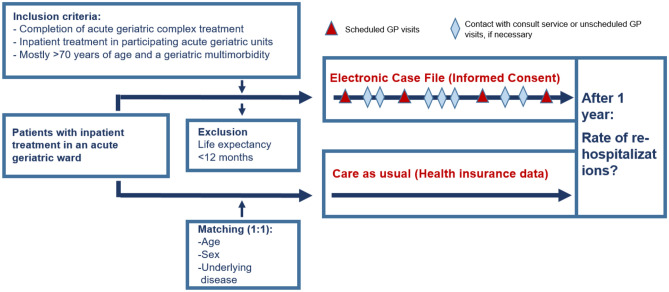
Description of the evaluation design.

After meeting the inclusion criteria and providing written consent to participate in the study, the supervising study center/discharging clinic entered examination findings, assessments, progress reports, medication schedules, and physician's letters into the ECF (Fig. 2). The ECF was easily accessible via the project homepage without the need for additional or specialized software. The corresponding access data were sent to the GP by the study center. The ECF was readily accessible to the general practitioner (GP) for further treatment. Therefore, the information could be viewed via the ECF before the patient's discharge and first visit to the GP. It is the responsibility of the GP to maintain the ECF for a year, which includes quarterly updates on the patient's status and a basic geriatric assessment. Any changes in the patient's status, initiated measures, and especially medication changes should also be documented. The hospital geriatrician reviewed and examined the current medication list together with a pharmacist specializing in geriatric medicine. If any further inquiries or possible action items arose, the medical consultation-service communicated with the GP either directly via a messenger function in the ECF or by telephone using specially set up numbers.

### Study outcomes and statistical analysis

The primary endpoint was the rehospitalization rate within one year after discharging from inpatient geriatric care. The project consisted of five survey time points: a baseline survey during inpatient treatment at one of the participating geriatric clinics, followed by four scheduled ambulant visits at the GP (HA1, HA2, HA3, HA4). Optional visits to the GP were also available throughout the follow-up period if necessary (Fig. 2). The rehospitalization rates of the control group were obtained from the BARMER W-DWH. Patients in the control group were matched 1:1 with patients within the geriatric new form of care. For this purpose, age (± 2 years), sex (exact) and underlying disease (ICD-10, two digits) were identified and matched in the BARMER (W-DWH). To address possible differences in severity/intensity of treatment, patients were additionally matched for the relative weight (also: case severity; factor ± 0.5). The relative weight is a multiplier (point value) of the base rate. It therefore indicates the factor by which the base case value is increased in the DRG system. A relative weight above 1 indicates a higher "case severity", as it is a more complex case than the average case.According to unpublished data from a health insurance company, approximately 70% of geriatric patients were rehospitalized at least once in the year following their inpatient stay with the previous standard form of care. The average re-hospitalization rate during that year was 1.85 (5411 cases / 1 year * 2931 insured persons). Assuming that the new form of care can reduce the rate to 1.30, 110 patients per care option would need to be included to hedge the difference between the two rates against chance with 0.05 alpha and 0.90 power. Therefore, a total of 220 patients would need to be included in the study and followed until the end. It is important to note that re-hospitalizations represent recurrent events and are likely to be correlated within a patient. Additionally, there may be a cluster effect within the hospital, so the number of cases must be increased to n = 275. Due to the advanced age of the patients, a relatively high drop-out rate of up to 50% was expected. This implies that 550 patients should be included.

To analyze the impact of the Covid-19 pandemic on hospitalizations, a multivariate Poisson regression was performed on the GerNe patient population. The GerNe patients were divided into three groups: group 1 consisted of patients with the complete observation period before the first lockdown (March 22, 2020); group 2 consisted of patients with observation times before and after March 22, 2020; and group 3 consisted of patients with all observation times after March 22, 2020. The regression analysis only included patients from groups 1 and 3 due to the lack of clear allocation for patients in group 2. Relevant confounders, such as age, gender, relative weight, and care level, were also adjusted for.

In addition, the GerNe project evaluated multiple secondary endpoints: level of care, home accommodation, vital status (death), medication (quality/quantity/changes), quality of life (SF-8 questionnaire), functional status (Barthel-Index) and health care utilization costs.

The level of care refers to the classification of individuals who require care due to health impairments or disabilities into different levels based on the extent of their needs. In the German care system, there are five care levels (1 to 5), with Care Level 1 representing the lowest and Care Level 5 representing the highest level of support needed. The higher the care level, the more comprehensive the care services that can be accessed. The level of care changes for GerNe patients were described objectively. Univariate comparisons using Chi^2^ tests were used to compare GerNe patients and control individuals.

The Short-Form Health Survey 8 (SF-8) was used to measure the quality of life. It consists of eight items that are transformed into eight domains: Physical functioning (PF), Physical role functioning (RP), Physical pain (BP), General health perception (GH), Vitality (VT), Social functioning (SF), Emotional role functioning (RE), and Mental well-being (MH). All domains were reported with values between 0 and 100, where higher scores indicated better health. The patients completed the SF-8 themselves at the beginning of the project and at the final GP visit (HA4). To evaluate quality of life, changes in individual domains and scales were examined and compared to a German age- and gender-adjusted norm population^14^. Mean value comparisons, effect sizes, and cross-tabulations were used to analyze changes over the course of the project.

The Barthel-index (BI) assesses a person's ability to perform ten basic activities of daily living, including eating, bathing, grooming, dressing, bowel and bladder control, toileting, transferring (e.g. from bed to chair), mobility and climbing stairs. Each activity is scored on a scale of 0 to 10, with higher scores indicating greater independence and functional ability. A maximum of 100 points can be achieved^15^. Changes over the course of the project were analyzed using means comparisons, effect sizes and paired t-tests.

The use of the new form of care was evaluated through the use of the consultation service. The evaluation of the consultation service was descriptive. For this purpose, the contacts (use of the messaging function) and the number of conversations (exchange with a patient with possibly several contacts) were presented in absolute figures.

The use of medical care services was documented in the case file. This data was identified and analyzed in the W-DWH for the BARMER control individuals.

The costs of hospital treatment (inpatient stays) were derived from the "basic case rate". The basic case rate is a financial indicator in the healthcare system that represents the monetary value of a standardized treatment case or a standardized service unit.

### Ethics

This study was performed in line with the principles of the Declaration of Helsinki. The affirmative vote was given by the Ethics Committee of the Rhineland-Palatinate Medical Association (Germany) on 18.06.2018 (No. 2018–13,347).

### Consent to participate

Informed consent was obtained from all individual participants included in the study.

## Results

The results of the GerNe project are also published in the G-BA results report (only available in German): https://innovationsfonds.g-ba.de/downloads/beschluss-dokumente/516/2024-01-19_GerNe_Ergebnisbericht.pdf^16^.

**Figure 3:**
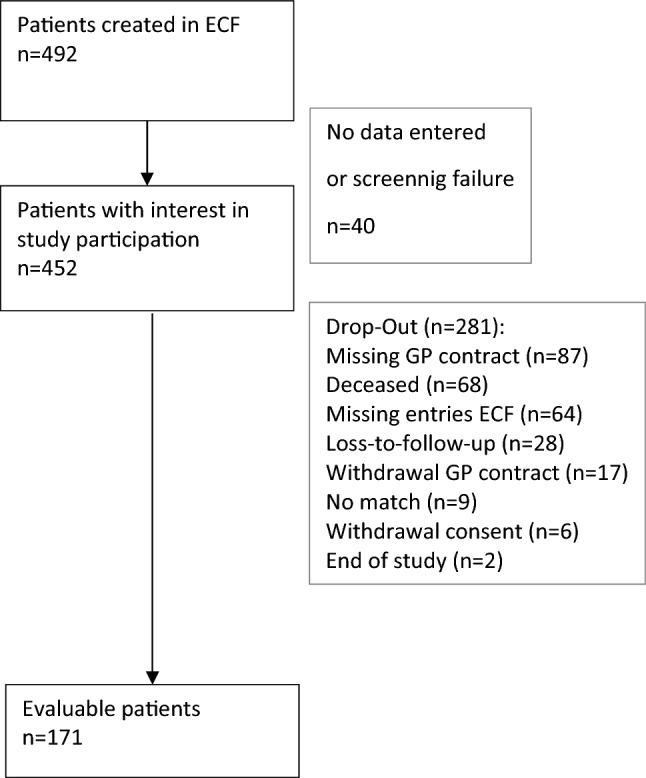
Flowchart of patient inclusion of GerNe patients.

The ECF recorded 492 patients, of whom 40 (8.1%) had no further data entered. A total of 171 cases were evaluated in this study (Fig. 3). Table 1 displays the characteristics of the study population. The dropout rate of 281 (62%) was within the acceptable range, but the reasons for dropout were somewhat surprising. Most reasons for dropping out were related to the GP, such as missing GP contracts (n = 87), withdrawal of a GP contract (n = 17), or leading to missing entries in the ECF (n = 64). The lower proportion of study discontinuations was attributed to the patients themselves, such as loss to follow-up (n = 28) or withdrawal of informed consent (n = 6). Initially, it was assumed that the drop-out rate would primarily be due to the death of elderly patients. However, the study population's average age was 84 years (SD 6.1). Only 68 patients (24.2%) died during the study period, which was below expectations.

**Table 1:** Baseline characteristics of GerNe-participants and drop-out.

Characteristics	Participants (n = 171)	Drop-Outs (n = 281)	p-value
**Age**			0.56
Mean (SD)	84.0 (6.1)	83.2 (6.9)	
Median (Q25; Q75)	83.7 (80.0; 88.2)	84.0 (79.8; 87.2)	
**Sex (% (n))**			0.38
Male	24.0 (41)	28.9 (81)	
Female	76.0 (130)	70.8 (199)	
Unknown	0	0.4 (1)	
**Relative weight**			0.08
Mean (SD)	2.3 (1.5)	2.1 (1.0)	
Median (Q25; Q75)	2.0 (1.8; 2.2)	1.9 (1.5; 2.1)	
**Marital Status (% (n))**			0.16
Single	3.5 (6)	8.5 (24)	
Married	35.1 (60)	32.0 (90)	
Widowed	52.6 (90)	47.0 (132)	
Divorced	5.3 (9)	6.4 (18)	
Unknown	3.5 (6)	6.0 (17)	
**Living environment (% (n))**			0.01
Alone	43.9 (75)	39.1 (110)	
With partner	33.3 (57)	28.5 (80)	
With children	6.4 (11)	12.5 (35)	
Nursing home	9.4 (16)	10.7 (30)	
Assisted living	7.0 (12)	3.9 (11)	
Unknown	0	5.3 (15)	
**Nursing service (% (n))**			0.001
Yes	34.5 (59)	25.6 (72)	
No	62.0 (106)	61.2 (172)	
Unknown	3.5 (6)	13.2 (37)	
**Aids (% (n))**			
Cane	24.6 (42)	17.1 (48)	0.07
Walker	75.4 (129)	63.7 (179)	0.01
Nursing bed	7.6 (13)	10.0 (28)	0.50
**Barthel-Index**			< 0.001
Mean (SD)	72.5 (20.9)	63.6 (25.4)	
Median (Q25; Q75)	80 (60; 90)	70 (45; 85)	

## Rehospitalization

The hospitalization rate for patients enrolled in the project was 83.1/100 patient years (PY), while the control group (CG) from BARMER data had a rate of 69.0/100 PY. The study found no statistically significant difference in the primary endpoint of the study between the intervention group and the control group (p = 0.15). Multivariate Poisson regression analysis showed no statistically significant association between GerNe participants and the control group (GerNe vs. CG: RR = 0.90; 95% CI [0.70; 1.17]). No statistically significant association was found for relative weight (RR = 0.99; 95% CI [0.89; 1.09]).

The study found that hospitalization rates were lower among older individuals (RR = 0.97; 95%CI [0.94;0.99]) and females (RR = 0.58; 95%CI [0.44;0.76]). During the observation period, it was observed that over 60% of patients in both groups did not require hospitalization again (GerNe: 63.7%; control group: 61.4%) (Table 2). It was suspected that this effect was linked to the Covid-19 pandemic. In a subgroup analysis, it was found that participants who had all observation times after March 22, 2020 (during the lockdown) had a lower re-hospitalization rate (RR = 0.52; 95% CI [0.30; 0.90]). No statistically significant associations were found for age (RR = 1.0; 95% CI [0.97; 1.04]), sex (RR = 0.93; 95% CI [0.54; 1.61]), relative weight (RR = 1.0; 95% CI [1.0; 1.0]), and nursing grade group (RR = 1.66; 95% CI [0.97; 2.87] (care group 0 vs 1/2); RR = 1.53; 95% CI [0.79; 2.95] (care group 0 vs 3/4/5)).

**Table 2:** Number of hospitalizations in the GerNe-participants and control group.

Hospitalizations (% (n)	GerNe	Control group
0	63.7 (109)	61.4 (105)
1	17.5 (30)	23.3 (40)
2	10.5 (18)	8.8 (15)
3	3.5 (6)	2.3 (4)
4	1.2 (2)	2.3 (4)
5	2.3 (4)	0.6 (1)
6	1.2 (2)	0
7	0	0.6 (1)
8	0	0.6 (1)

## Utilization of the consultation service

Out of the total number of 171 participants, the ECF was used to initiate 195 contacts. The data collected on the use of the consultation service showed that the clinic or the supervising study center initiated contact via the ECF in approximately 70% of all cases (n = 195). Approximately 50% of the entries were related to organizational matters, such as reminders in the ECF, technical implementations in the ECF or information about drop-outs. Fifty percent of the inquiries or contacts received through the ECF were related to improvement of patient heath care. The majority of these inquiries (88 vs. 8 inquiries) were initiated by the clinics. The inquiries were mainly about drug treatment and care. In approximately 35% of the cases related to drug treatment, a change in medication was documented in the ECF following contact. However, in the majority of cases, no changeover took place or was not documented in the ECF. It is unclear whether medication changes were adopted but not documented in the ECF, as there is no data available on the use of the medication plan. The number of contacts, approximately 195, initiated via the ECF should be considered relatively low. It appears that the consultation service, which is a central element of the intervention, was utilized less than anticipated. In addition to the documented consultation via the ECF, there were also contacts between the clinic and the GP via email or phone that could not be clearly accounted for.

## Barthel-Index (BI) and SF-8- questionnaire

A survey in the sense of a progression parameter was only possible for the BI and SF-8-Questionnaire in the intervention group, since these data were not available from the BARMER Science Data Warehouse.

At discharge from the clinic, the mean BI was 72.5 points (SD: 20.9). Over the course of one year, there was only a very slight deterioration to 70.1 (SD 22.9) points, which corresponded to an effect size of -0.1 (95% CI [-0.2; 0.1]) and thus resulted in no effect. This difference was not statistically significant. This indicates that there was little to no deterioration in self-help ability approximately one year after discharge from the geriatric clinic. In view of the highly aged patient population, this is to be regarded as positive.

The SF-8 Survey categories social functioning (SF) and vitality (VT) showed clear and statistically significant deterioration during the observation period. In the other categories, however, there were no significant changes.

## Costs

The average cost per hospitalization in the control group was used to determine the cost of hospitalization. These averaged € 6655 per hospitalization (median: € 6662; Q25: € 3136; Q75: € 7915). The average cost per person-year in the GerNe group was thus €5748, while the cost in the control group was €4526 per person-year (p = 0.55). As it is difficult to estimate the costs of a possible implementation of the case record in regular care, these costs were not included in the cost comparison as one-time investment costs.

## Discussion

The GerNe project was well accepted by the patients; conversely, the participation of the GP did not meet the previous expectations. Regrettably, the consultation service and the ECF, which serves as a central communication platform, were not utilized by the GPs as intended. The documented communication between the GPs and the geriatric clinic mainly pertained to organizational matters, such as reminders of missing entries, and was mostly initiated by the clinic. There was no significant medical exchange between the parties. This leads to the assumption that the intervention already had no effect, so it is not surprising that there was no statistically significant difference between the intervention group and the control group in terms of rehospitalization rates. Regardless of the difficulties associated with inpatient recruiting, a sufficient data set could be collected in both groups. The non-participation of GPs in studies is generally a problem^17^.The primary reason for non-participation is typically a lack of time. The reason of 'lack of interest' is usually of secondary importance^18^. International studies have also documented the issue of non-participation by GPs^19,20^. The recruitment of GPs was more successful and resulted in more frequent participation when medical colleagues and study assistants were involved^20^. This phenomenon was also observed in the GerNe project. However, despite the participants' willingness to participate, they did not end up using the consultation service and the ECF. The limited time resources of the GPs, who were additionally burdened by the COVID-19 pandemic during the course of the study, were certainly one reason for this. According to an online survey of GPs in Germany, the pandemic has led to an increase in workload and consultation hours, resulting in less time available for treating patients with chronic diseases or cancer screening examinations^21^. The COVID-19 pandemic had also an impact on hospitalization rates in Germany. GerNe-participants who were observed after March 22, 2020, when the lockdowns began, had a statistically lower rate of hospitalization. This association has also been reported in the literature^22^. During the pandemic, patients have also refrained from visiting their GP^23^.

If there was communication via the case file in the sense of the project, this concerned questions or comments regarding the medication taken. This is not surprising, as the topic of polypharmacy and the adaptation of medication for older patients to their individual needs is an essential aspect of daily work in geriatrics^24^. In Germany, approximately 42% of individuals aged 65 and older experience polypharmacy, defined as taking five or more active substances^25^. This percentage increases to around 50% for patients aged 85 and older^26^. The risk of adverse drug reactions (ADRs) and drug interactions (DDIs) increases with the number of medications taken. In the GerNe project, medication and changes to it were constantly monitored by the consultation service, which also included the expertise of a pharmacist specializing in pharmacology for the elderly. This intervention is expected to decrease the incidence of adverse drug reactions (ADRs) and drug-drug interactions (DDIs). There are already programs in Australia in which pharmacists work with GPs to review complex medication regimes, identify causes of drug-related problems, and recommend solutions to prevent or resolve them^27^.

The study shows the difficulty of establishing an ECF involving various stakeholders. This is also evident in the introduction of the electronic patient record (ePA) in Germany. More than 2 years after its introduction, it is still only being used by approximately 1% of those with statutory health insurance^28^. The electronic patient record (ePA) can be particularly beneficial for complex, multimorbid patients as it centralizes their diagnoses, findings, and medications. This is especially useful for the treatment of geriatric patients. Additionally, algorithms can be used to perform a medication check and provide information on potential drug-drug interactions (DDIs) or potentially inappropriate medications (PIMs) for older patients. The algorithm has already undergone testing for the FORTA list (Fit for The Aged)^29^. The distinctive feature of the FORTA list is the evaluation of drugs on the basis of the available diagnoses, taking into account previous findings and life circumstances. It is evaluated whether the benefit of a drug is very advantageous/beneficial in the elderly patients or whether its use in these patients should be viewed critically or avoided^30^.

However, the benefits of e-health components are widely recognized and have been particularly evident during the COVID-19 pandemic. As of March 30, 2021, the Federal Joint Committee (G-BA) has included telemonitoring of heart failure patients as a standard method for treating and examining these patients. Studies have demonstrated that the use of such components results in reduced mortality and cardiovascular morbidity^31^. Data was collected using invasive methods such as implantable cardioverter-defibrillators (ICDs) or noninvasive methods such as smart scales, blood pressure monitors and pulse oximeters. The collected data were evaluated at a telemetry center. In the context of the GerNe project, a transfer to an ECF would also be possible. In addition to using of devices to transmit vital signs to the attending physician or an ECF, video consultations have become established in the outpatient sector. Although digital tool cannot replace direct doctor-patient contact, they have made a valuable contribution to care during the pandemic and helped to reduce the risk of infection^32^. We are convinced that geriatric patients in particular can benefit from these "new" digital possibilities. In this context, previous studies have already emphasised the positive effects of geriatric co-management on the discharge process of older people. In line with the TCM, support is provided through structured care by trained professionals^10,11^. The Innovation Fund project "TIGER" was also based on TCM. Specially trained "pathfinders" accompanied the discharge process of geriatric patients. During the inpatient stay, a care and support plan for the time after discharge was developed together with relatives, doctors, nursing staff and social services. After discharge, regular visits to the patient's home or telephone contact and exchange of information took place in a structured way between the different health care providers. The care and support plan was adapted as needed and coordinated with other stakeholders. This project was also unable to show a reduction in rehospitalizations, but positive experiences were also reported here, so that the project of care was continued in form of a 6-week follow-up at home^33^.

## Conclusion

Several studies have highlighted the significance of a supported transition from inpatient care to subordinate settings, such as the home environment or a nursing facility^34–36^. There are many approaches to reducing hospital readmissions. As with daily work in geriatrics, an interdisciplinary approach has proven successful in this regard, involving various professional groups^8^ in the discharge process while considering the personal resources^37^ of patients . Even though the new form of care did not show a difference from standard care in terms of a reduction in hospital readmissions, we believe that digital networking between the treating geriatrician in the hospital and the primary care physician will lead to better care for geriatric patients.

## Limitations

The validity of the study is certainly susceptible to confounding variables due to the small intervention group and the high number of dropouts.

Only one health insurance company, BARMER health insurance, was involved in the project. This meant that only patient data from this health insurance company could be used for the control group. This could lead to some kind of bias, as the patient populations of the different health insurers differ in terms of demographic and other personal characteristics. As the differences in the patient populations are not clearly known, it is not possible to quantify how these differences might influence the results of this study. It should also be noted that some data in the area of functionality and quality of life could not be collected because the questionnaires were not returned in full. Geriatric patients, in particular, may have difficulty completing the questionnaires in general. During the COVID-19 pandemic period, the resources available to healthcare providers to support and complete the questionnaires were even more limited. This lack of data affected the accuracy of the matching results, which in turn may have affected the outcome of this trial. Due to the study design using secondary data from an health insurnace randomasition of study particpants was not possible. Furthermore, it is not clear whether all hospitalisations were correctly recorded by the participating GPs. It cannot be ruled out that hospitalisations were not recorded by the GP, which would have an impact on the rate of rehospitalisation and thus on the primary outcome.

In addition, it is not clear whether a positive effect of the study would have been detectable if the GPs had been more actively involved and the Covid-19 pandemic had not influenced the study. It would have been desirable to integrate the ECF into the GPs' software to increase the willingness to participate, but this was not possible due to data protection issues.

## Data Availability

The datasets used and/or analyzed during the current study available from the corresponding author on reasonable request.
